# A multimodal study regarding neural correlates of the subjective well-being in healthy individuals

**DOI:** 10.1038/s41598-022-18013-1

**Published:** 2022-08-11

**Authors:** Hye-Yeon Jung, Chongwon Pae, Iseul An, Minji Bang, Tai Kiu Choi, Sung Joon Cho, Sang-Hyuk Lee

**Affiliations:** 1 Department of Psychiatry, CHA Bundang Medical Center, CHA University, 59 Yatap-ro, Bundang-gu, Seongnam-si, Gyeonggi-do 13496 Republic of Korea; 2 Department of Psychiatry, Kangbuk Samsung Hospital, Sungkyunkwan University School of Medicine, 29 Saemunan-ro, Jongno-gu, Seoul, 03181 Republic of Korea

**Keywords:** Cognitive neuroscience, Emotion

## Abstract

Although happiness or subjective well-being (SWB) has drawn much attention from researchers, the precise neural structural correlates of SWB are generally unknown. In the present study, we aimed to investigate the associations between gray matter (GM) volumes, white matter (WM) microstructures, and SWB in healthy individuals, mainly young adults using multimodal T1 and diffusion tensor imaging studies. We enrolled 70 healthy individuals using magnetic resonance imaging. We measured their SWB using the Concise Measure of Subjective Well-Being. Voxel-wise statistical analysis of GM volumes was performed using voxel-based morphometry, while fractional anisotropy (FA) values were analyzed using tract-based spatial statistics. In healthy individuals, higher levels of SWB were significantly correlated with increased GM volumes of the anterior insula and decreased FA values in clusters of the body of the corpus callosum, precuneus WM, and fornix cres/stria terminalis. A correlational analysis revealed that GM volumes and FA values in these significant regions were significantly correlated with severity of psychological symptoms such as depression, anxiety, and quality of life. Our findings indicate that GM volumes and WM microstructures in these regions may contribute to SWB, and could be the neural basis for psychological symptom severity as well as quality of life in healthy individuals.

## Introduction

Happiness, or subjective well-being (SWB), is a multidimensional construct that concerns optimal experience and functioning. It has several components, including a cognitive aspect of life satisfaction, an affective aspect of the presence of positive emotions and mood, and the absence or reduction of negative emotions and mood, together often summarized as happiness^1^.

SWB plays a considerable role in enhancing individuals’quality of life, such as success in psychological health, work, and interpersonal relationships. Low SWB acts as a risk factor for depression^2^ as well as anxiety^3^, and poor mental health^4^. Individuals reporting greater levels of SWB showed several positive aspects of cognitive and motivational processes, such as encoding relatively more positive than negative events into memory, perceiving and interpreting life circumstances in positive ways, being less sensitive to negative feedback, and excessive self-focused cognition (rumination)^5,6^. Additionally, higher SWB was associated with stronger emotional regulation abilities, resilience to stress, and mindfulness^7^. These characteristics affect how individuals manage stressful situations and respond effectively by enhancing well-being^8^. Furthermore, a review study indicated that SWB is associated with cognitive control processes and a flexible brain network that responds to perpetually shifting contextual task demands^9^.

Several functional brain magnetic resonance imaging (fMRI) studies have reported that default mode network (DMN) and salience network may have implications for SWB. fMRI studies have shown that individuals with higher SWB show less activation and decreased functional connectivity in the default mode network (DMN) nodes, such as the precuneus and posterior cingulate cortex, which indicates a flexible brain network^10,11^. Hence, reduced functional connectivity is correlated with less rumination and flexible affective processing. Furthermore, evidence has shown a positive correlation between social well-being and the fractional amplitude of low-frequency fluctuations in the salience network including the right anterior cingulate cortex and insula^12^. Another functional neuroimaging study reported that SWB negatively correlates with functional connectivity between the salience network and DMN^13^. Structurally, previous MRI studies have consistently found that SWB in individuals is related to brain areas associated with emotional regulation, specifically, the insula^14^, anterior cingulate cortex^15,16^, dorsolateral prefrontal cortex^17^, and orbitofrontal cortex^18^. In particular, a previous MRI study indicated that healthy individuals with larger gray matter (GM) volumes in the insula were correlated with eudaimonic well-being and polygenic scores of SWB^19,20^. However, whether and how general SWB-related GM and white matter (WM) brain regions are related to other psychological characteristics, such as quality of life and psychiatric symptoms, has never been investigated in healthy individuals. In addition, the neural structural correlates of SWB in multimodal neuroimaging studies and the relationship between WM and GM regions related to SWB are not yet known. Taken together, these results suggest that elevated SWB among healthy individuals may be related to brain regions associated with the DMN and emotional regulation. Therefore, it is needed to investigate whether GM volumes and WM microstructures can be associated with SWB, as well as to reveal their inter-relationships among the neural correlates of GM and WM regions significantly correlated with SWB in healthy individuals.

In the present study, we investigated neural correlates such as GM volumes and WM microstructures related to SWB, three SWB subscales, and their associations with the severity of symptoms and mental health in quality of life in healthy individuals. We also examined the inter-relationships among neural correlates of the GM and WM regions that were significantly associated with SWB. We hypothesized that (1) GM volumes and WM microstructures in the insula and DMN-related brain WM regions in healthy individuals would be related to SWB, (2) there could be a significant association between SWB-related GM and WM regions and the severity of symptoms such as depression, anxiety, and quality of life, and (3) there would be an inter-relationship among neural correlates of GM and WM regions significantly associated with SWB.

## Results

### Demographic and clinical characteristics of study participants

Table 1 summarizes the demographic and clinical characteristics of the study participants.

**Table 1:** Demographic and clinical characteristics of study participants.

	HIs (n = 70)
**Sex**	
Male, *n* (%)	32 (45.7%)
Female, *n* (%)	38 (54.3%)
Age (years, mean ± SD) (age range)	36.6 ± 8.86 (20–61)
Education (years, mean ± SD)	17.22 ± 2.04
Intracranial volume (ml, mean ± SD)	1529.77 ± 131.3
**Religion**	
Existed (*n*)	31
None (*n*)	20
**Monthly income**	
Above 1800 $USD (*n*)	49
Below 1800 $USD (*n*)	2
**Marital status**	
Living with partner (*n*)	29
Living without partner (*n*)	28
**Job**	
Existed (*n*)	50
None (*n*)	7
**COMOSWB, total scores (mean ± SD)**	20.59 ± 7.17
Life satisfaction (*n*)	15.47 ± 2.44
Positive emotion (*n*)	14.59 ± 3.05
Negative emotion (*n*)	9.47 ± 3.11

*HI* healthy individual, *SD* standard deviation, *COMOSWB* concise measure of subjective well-being.

### Associations between subjective well-being and the gray matter volumes in healthy individuals

**Figure 1:**
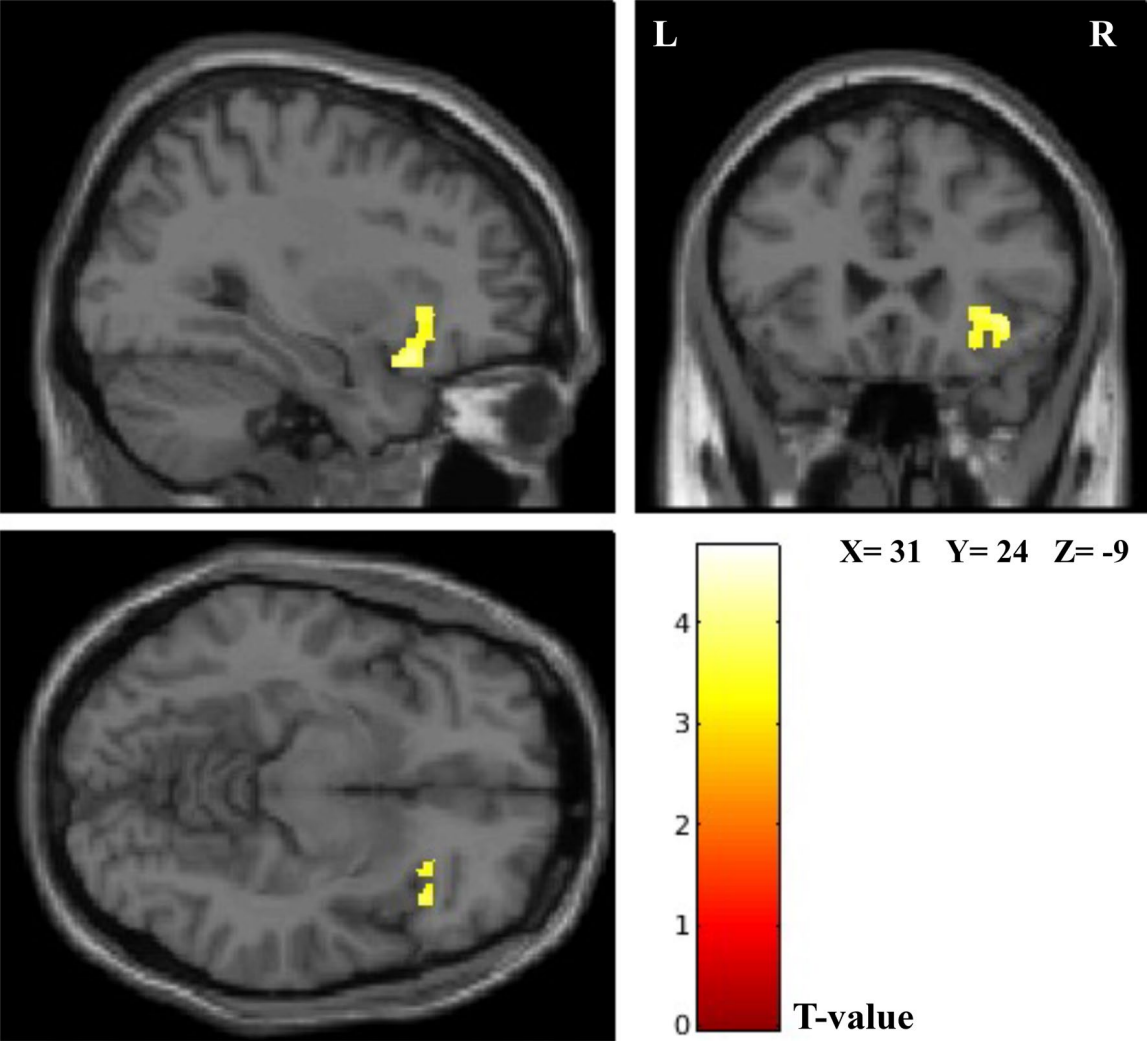
Regions of significant positive association between gray matter volume in the anterior insula and COMOSWB total scores in healthy individuals are overlaid on a T1weighted standard MNI. The red-to-yellow color scale denotes the T-value (p FWE < 0.05 at cluster level; k > 400 for visualization purposes; k = cluster size (number of voxels). *R* right, *L* left, *MNI* Montreal Neurologic Institute, *COMOSWB* concise measure of subjective well-being.

Whole brain analysis of voxel-based morphometry (VBM) showed a significant positive correlation between GM volumes in the anterior insula (AI) and the Concise Measure of Subjective Well-Being (COMOSWB) total scores (p family-wise error (FWE) = 0.017 at the cluster level) (Fig. 1). After the analysis of covariance for age, sex, and total intracranial volume (TIV), the association remained significant. In addition, life satisfaction subscale scores were positively correlated with AI (p FWE = 0.02, at the cluster level). There were no significant associations between gray matter volume and other positive and negative emotion subscale scores.

### Correlations of gray matter volumes in anterior insula with symptom severities and quality of life in healthy individuals

**Figure 2:**
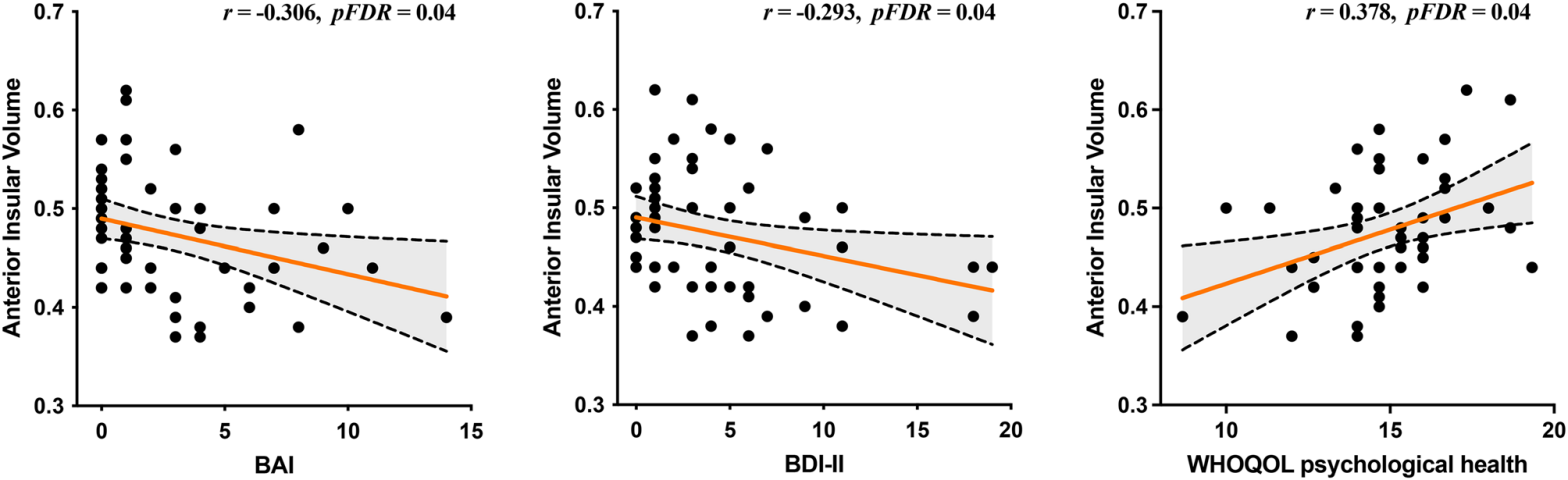
Pearson correlation analyses were performed to determine the relation between the adjusted gray matter volume values in the anterior insula with the other psychological characteristics. BAI, Beck-Anxiety Inventory; BDI-II, Beck-Depression Inventory-II; WHOQOL, World Health Organization Quality of Life. The red line in each graph represents the regression line, and the back dotted line with shaded area indicates the 95% confidence intervals (CI).

The results of Pearson correlation analysis showed that the adjusted GM volume values of the SWB-related AI were significantly positively associated with the World Health Organization Quality of Life (WHOQOL) psychological health subscale scores (r = 0.378, p false discovery rate (FDR) = 0.04). Subsequently, adjusted GM volume values in the AI were negatively associated with the total scores of the Beck Anxiety Inventory (BAI) and Beck Depression Inventory Second Edition (BDI-II) (r = − 0.306, p FDR = 0.04, and r = − 0.293, p FDR = 0.04, respectively) (Fig. 2).

### Associations between white matter microstructures and subjective well-being in healthy individuals

**Figure 3:**
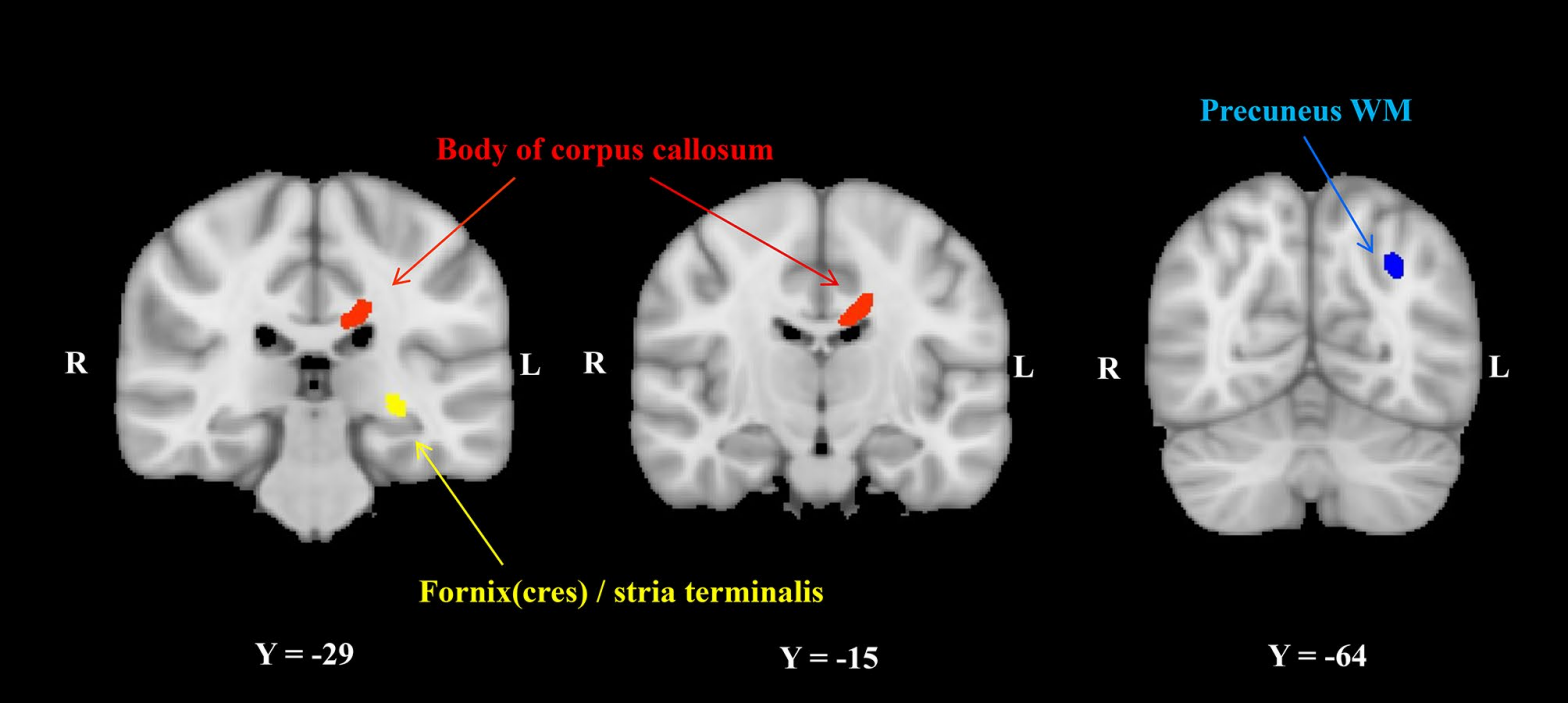
Regions of significant negative correlation between fractional anisotropy values and the COMOSWB total scores in healthy individuals (threshold-free cluster enhancement, p < 0.05 familywise error corrected) are shown in yellow, red and blue. For better visibility, the results are thickened using the “tbss-fill’ command. Number of permutations was 5000. *WM* white matter, *R* right, *L* left, *COMOSWB* concise measure of subjective well-being.

The voxel-wise whole-brain analysis demonstrated that the COMOSWB total scores were significantly and negatively correlated (TFCE-corrected p < 0.05) with the fractional anisotropy (FA) values in clusters of the body of the corpus callosum (CC), fornix cres/stria terminalis (FX/ST), and precuneus WM (Fig. 3). After controlling for sex and age as covariates, the findings remained unchanged. In addition, there was a significant negative correlation between positive emotion subscale scores and the posterior corona radiata, superior longitudinal fasciculus, and splenium of the CC (TFCE-corrected p < 0.05). No significant correlations were found between FA values, life satisfaction, and negative emotion subscale scores.

### Correlations between fractional anisotropy values in white matter regions significantly correlated with symptom severity and quality of life in healthy individuals

The mean FA values of the precuneus WM were positively with the total BAI and BDI-II scores (r = 0.28, p FDR = 0.038 and r = 0.354, p FDR = 0.012, respectively). The mean FA values extracted from a significant cluster of FX/ST showed a positive correlation with the total BAI scores (r = 0.336, p FDR = 0.017). The WHOQOL subscale scores of psychological health showed significant negative correlations with the mean FA values extracted from the significant cluster of body of CC, FX/ST, and precuneus WM (r = − 0.487, p FDR = 0.004, r = − 0.531, p FDR < 0.001, and r = − 0.472, p FDR = 0.004, respectively). In addition, the WHOQOL social health subscale scores were significantly and negatively correlated with the mean FA values extracted from a significant cluster of the FX/ST and the precuneus WM (r = − 0.454, p FDR = 0.004 and r = − 0.415, p FDR = 0.01, respectively).

### Voxel-wise correlation between gray matter volumes and white matter microstructures associated with subjective well-being

**Figure 4:**
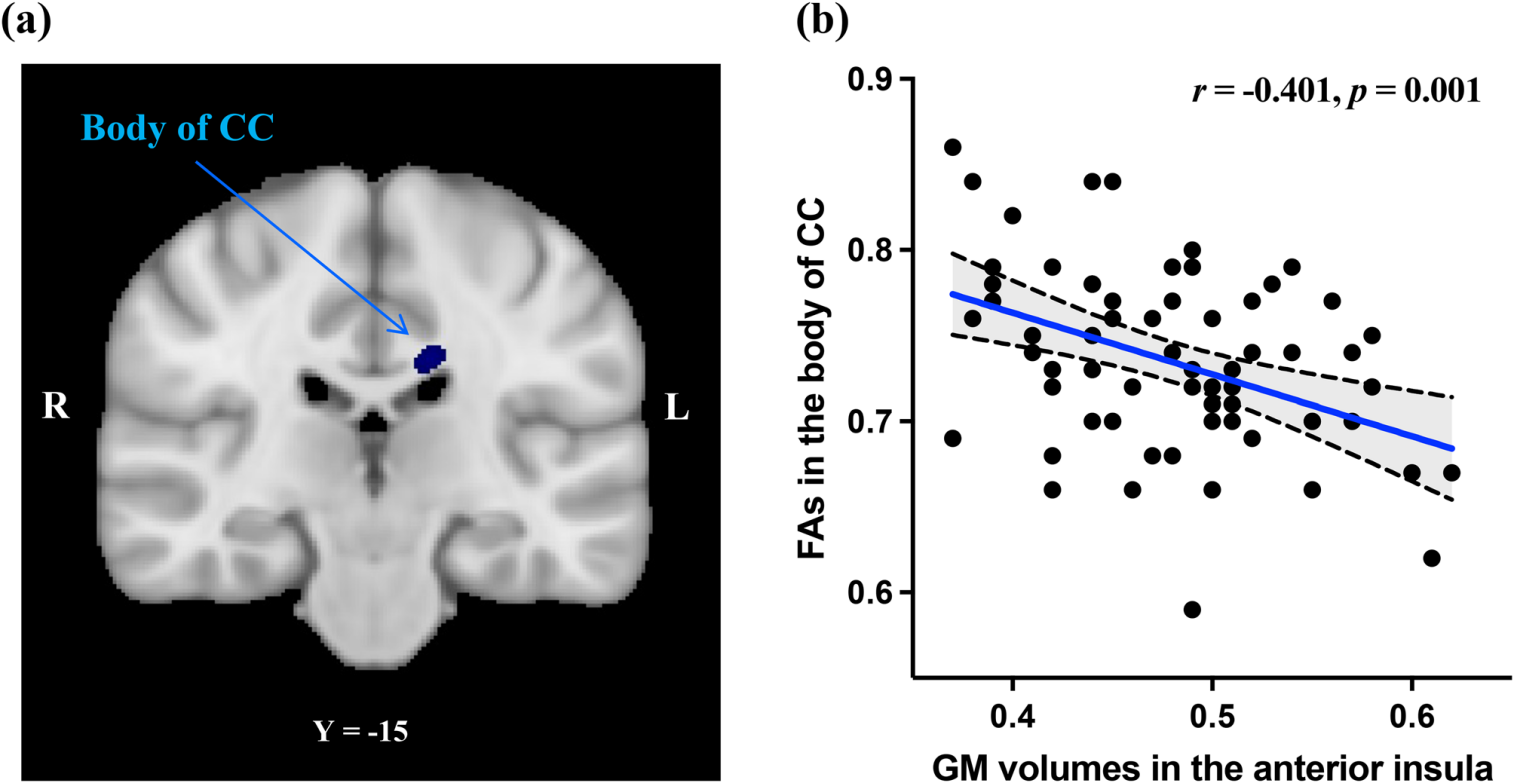
(**a**) Shows a region of significant negative correlation between adjusted GM volumes in the anterior insula and fractional anisotropy values in the body of CC associated with subjective wellbeing in healthy individuals (threshold-free cluster enhancement, p < 0.05 family-wise error corrected). For better visibility, the results are thickened using the “tbss-fill’ command. (**b**) Shows scatter plots of the negative correlation between GM volumes and fractional anisotropy values extracted from each subject in the significant regions indicated in. The blue line in each graph represents the regression line, and the back dotted line with shaded area indicates the 95% confidence intervals (CI). *CC* corpus callosum, *GM* gray matter, *R* right, *L* left.

The adjusted GM volume values in the AI were significantly negatively correlated (TFCE-corrected p < 0.05) with the FA values in the significant clusters of the body of the CC (Fig. 4). No significant correlations were found between the adjusted GM volume values in the AI and other significant WM clusters such as FX/ST and precuneus WM.

## Discussion

To the best of our knowledge, this is the first multimodal T1 and diffusion tensor imaging (DTI) study to reveal the relationship between SWB and GM volumes in the AI and WM microstructures in the FX/ST, precuneus WM, and body of the CC in healthy individuals. In particular, further analysis revealed that the GM volumes in the AI were correlated with the FAs in the body of the CC, which may show the inter-relationships between WM and GM for contributing to SWB. We demonstrated that higher GM volume values in the AI and lower FA values in the FX/ST, precuneus WM, and body of the CC were correlated with higher SWB in healthy individuals. Furthermore, adjusted GM volume and FA values in these significant regions were significantly associated with quality of life and severity of symptoms, such as depression and anxiety.

The present study found that, among healthy individuals, higher GM volume values in the AI were related to higher COMOSWB total scores. Although in several previous neuroimaging studies, the insula was mainly considered as a region functionally and anatomically related to only social and eudaimonic wellbeing, not general SWB^12,19^, relationship between the total scores of COMOSWB, which reflects general SWB and GM volumes of the AI, has not been shown yet. AI, which has reciprocal connections to limbic regions, plays a crucial role in interoception and emotional awareness^21^. According to a functional neuroimaging study, AI, a crucial region in salience network integrates information from cognitive, emotional, and affective processes^14^. Hence, AI supports subjective feeling states and regulates the introduction of subjective feelings into cognitive and motivational processes^22^. Our findings may indicate that accurate interoception and integration of emotional and affective processes from increased GM volumes in the AI is a critical component of SWB.

Furthermore, our findings revealed a positive correlation between GM volume in the AI and the life satisfaction subscale scores of the COMOSWB. Life satisfaction is an essential component of SWB and can influence a global judgment of quality of life according to an individual’s own needs and expectations^23^. Higher life satisfaction reflects greater flexibility, more positive self-cognition, relative insensitivity to negative stimuli, and reaction to more positive contexts^24^. Our findings are partly supported by previous studies on diffusion microstructure reporting significant associations between the frontoinsula and life satisfaction^23,25^This result implies that life satisfaction, the components of SWB caused by regulating of positive stimuli and emotions, are associated with increased GM volumes in the AI.

We also revealed negative correlations between adjusted GM volumes in the AI correlated with SWB and the total BDI-II and BAI scores. Subsequently, we found that the adjusted GM volumes in the AI were positively correlated with the psychological health subscale scores of the WHOQOL. Our findings are consistent with those of previous studies reporting AI to be associated with quality of life and the severity of symptoms such as depression, anxiety^26,27^. Taken together, less severe symptoms and a higher quality of life could be associated with higher SWB in healthy individuals.

The findings of our study suggest that lower mean FA values in the body of the CC, FX/ST, and precuneus WM were associated with higher COMOSWB total scores. FA, which represents WM microstructures, is a sensitive measure of the degree of water diffusion along the WM tracts^28^. Our results are partly in line with a prior study indicating that meditators with improved emotional regulation exhibited relatively less activation in the hippocampal formations, amygdala, and primary nodes of the DMN^29^. Furthermore, functional connectivity in the DMN regions was correlated with reduced rumination^30^, which can lead to higher SWB. These previous studies support our results, reporting that higher levels of SWB could be characterized by decreased WM connectivity in the FX/ST related to the hippocampal formations and amygdala^31,32^, and the CC and precuneus related to the DMN^33^.

The CC, the largest fiber bundle connecting the two cerebral hemispheres, is widely regarded as an important region responsible for interhemispheric integration and information processing of input and output signals to facilitate coordination of thoughts and behaviors^34,35^. In a previous study, interhemispheric inhibition mediated by the CC was related to emotional functioning, such as regulating emotions and recognizing emotional state^36^. Although CC has not been related to SWB in previous neuroimaging studies, CC was found to be crucial for resilience as an important predictor for enhancing SWB^37^. Functionally, lower FA values in the body of the CC may lead to enhanced emotional functioning related to SWB in healthy individuals.

The FX is an important WM tract bundle that is located underneath the CC, connects the hippocampus and septum, and plays a crucial role in the formation and consolidation of declarative memories^31^. FX is a part of the Papez circuit, which is an important structure of the limbic system and is involved in the regulation of emotions by higher-order frontal cortical brain regions^38^. The ST is the WM tract bundle, which is thought to connect the septum and amygdala and participates in cognition and emotional regulation with the FX and cingulum bundle from the limbic system^32^. As demonstrated by our findings, adaptive cognition and emotional regulation by the microstructure of FX/ST may be associated with higher SWB.

The functional core of the DMN^39^, the precuneus, is part of the flexible affective workspace and is functionally and structurally connected to the medial prefrontal cortex and posterior cingulate cortex in the DMN^40,41^. A previous study reported a positive relationship between FA and functional connectivity in the DMN in healthy subjects^42^. These studies have shown that precuneus WM affects functional connectivity in the DMN. Our findings are a continuation of a previous functional neuroimaging-based study^10^ indicating that higher levels of SWB lead to a stable DMN. Thus, hyperconnectivity and hyperactivation of the DMN are associated with higher levels of rumination about self-feelings, thoughts, and emotions, which leads to lower levels of SWB^10^. Moreover, the precuneus is considered to play a crucial role in integrating different types of information and converting it into subjective happiness in a previous study regarding the precuneus as the structural correlate of happiness in healthy individuals^43^. Our findings suggest that decreased FAs in lower levels of rumination and adaptive integration of internal and external information related to emotional regulation caused by reduced FAs in the precuneus may contribute to higher SWB.

In the subscale analysis, we also found a significant correlation between the positive emotion subscale scores and the FA values of the posterior corona radiata, superior longitudinal fasciculus, and splenium of the CC. Experiencing positive emotions benefits psychological and physical well-being in intersecting ways, including modulating neurophysiological correlates within the central and peripheral nervous systems, and is associated with the brain networks implicated in cognitive control and flexible affective processing^9^. The posterior corona radiata, superior longitudinal fasciculus, and splenium of the CC are included in the main WM regions related to the DMN as part of the flexible brain network^33^. Our findings indicate that decreased FA values in these regions related to the DMN may affect positive emotions through flexible affective processes and adaptive cognitive control.

Furthermore, our results demonstrated that WM microstructures in the FX/ST and precuneus related to SWB was positively correlated with the severity of psychological symptoms such as depression and anxiety. BAI scores were correlated with the extracted FA values of the FX/ST and precuneus WM. Moreover, BDI-II scores were positively correlated with the precuneus WM. These results are consistent with the observations in previous studies showing that the precuneus is associated with the pathophysiology of depression and anxiety^44^ and that the FX/ST microalterations may be related to the septum’s effect on anxiety regulation^45^. Our findings thus indicate that WM microstructures in the FX/ST and precuneus WM contribute to the lower severity of psychological symptoms.

Interestingly, correlation analysis showed that the decreased FA values in the body of the CC, FX/ST, and precuneus WM related to SWB were associated with the social and psychological health subscale scores of WHOQOL. We observed a negative relationship between FA values in the body of the CC, FX/ST, and precuneus WM and psychological health, as well as between the FX/ST and precuneus WM and social health. This is partly supported by a previous study reporting that DMN was associated with psychological and social health in terms of quality of life^46^. In the present study, the association was replicated only in the body of the CC, FX/ST, and the precuneus WM related to DMN, which are more specific areas than those in previous studies.

In addition, our further analysis revealed that the GM volumes in the AI were correlated with the FAs in the body of the CC related to SWB. This finding supports a previous study showing that AI is connected with CC^47^. Our findings also imply that GM in the AI and WM in the CC can play an important role, interacting with each other in the generation of SWB.

This study has a few limitations that should be interpreted with caution. First, since the sample size was relatively small, the association between SWB and the insula, body of CC, FX/ST, and precuneus WM should be confirmed using a larger sample. Second, all measures were based on self-report, which relied on the awareness of individuals, and the results may be affected by response bias. Nevertheless, all assessments were objective indicators that had already been repeatedly used and verified.

In conclusion, our study demonstrated increased GM volumes in the AI and decreased WM connectivity of the body of the CC, FX/ST, and precuneus in healthy individuals with higher SWB, its association with symptom severity and quality of life, and the inter-relationship between GM volume and WM microstructure. The present study results suggest that these regions can be considered potential predictive markers for SWB, as they are replicated repeatedly and the significance between symptoms is verified.

## Methods

### Participants

We recruited 70 healthy individuals with MR structural and diffusion images from the local community through online and print advertisements in the Department of Psychiatry of CHA Bundang Medical Center (Seongnam, Republic of Korea) between January 2014 and September 2021. All participants were right-handed and between 17 and 65 years of age; left-hand individuals were excluded as assessed using the Edinburgh Handedness Inventory^48^. Exclusion criteria for all participants were as follows: current or history of major medical diseases, such as diabetes mellitus, hypertension, substance disorders, intellectual disability, and psychiatric disorders. All study procedures adhered to the Institutional Review Board (no. 2019-05-030, 2021-03-001) of CHA Bundang Medical Center in accordance with the latest version of the Declaration of Helsinki and principles of Good Clinical Practice. Written informed consent was obtained after an explanation of the study procedures and purpose.

### Assessments

SWB was assessed using the Korean version of the COMOSWB, which revealed high reliability (Cronbach’s α = 0.86)^49^ and a high value for evaluating general SWB. The survey consists of nine self-reporting questionnaires assessing the following three domains: life satisfaction, which evaluates personal, relational, and collective life satisfaction; positive emotion and negative emotion, which consist of items representing high, medium, and low levels of emotional arousal, and each domain consists of three questionnaires. It was rated on a scale ranging from *strongly disagree* (1) to *strongly agree* (7) during the past month. The total scores were defined by subtracting negative emotion subscale scores from the sum of life satisfaction subscale scores and positive emotion subscale scores and range from − 15 to 39.

To evaluate the severity of anxiety and depressive symptoms in participants, the BAI^50^ and BDI-II^51^ were used. The scales showed high internal consistency (Cronbach’s α = 0.92 for BAI and Cronbach’s α = 0.91 for BDI-II)^52,53^. Additionally, the WHOQOL-BREF was administered to assess the participants’ quality of life. The WHOQOL-BREF contains 24 items in the four domains of physical, psychological, social, and environmental health. In addition, it has two facets: evaluating the overall quality of life and general health. The instrument domains have high internal consistency (Cronbach’s α = 0.90) and a validity^54^. The raw scores were calculated and converted into transformed scores.

### Neuroimaging data acquisition

All participants underwent brain MRI at baseline using a 3.0-Tesla GE Signa HDxt scanner (GE Healthcare, Milwaukee, WI, USA), which consisted of an 8-channel phase-array head coil. All MRI scans were acquired at the CHA Bundang Medical Center at CHA University.

The parameters for a three-dimensional T1-weighed fast spoiled gradient-recalled echo sequence (repetition time = 6.3 ms; echo time = 2.1 ms; flip angle = 12°; field of view = 256 mm; matrix = 256 × 256; voxel size = 1 × 1 × 1 mm^3^) was used.

Diffusion-weighted imaging (DWI) were acquired using an echo planar imaging (EPI) sequence using the following parameters: repetition time, 17,000 ms; echo time, 108 ms; field of view, 240 mm; acquisition matrix, 144 × 144; slice thickness, 1.7 mm; and voxel size, 1.67 × 1.67 × 1.7 mm^3^. A double-echo option was implemented to reduce the eddy current-related distortions. To reduce the effects of EPI spatial distortions, an 8-channel coil and an array of spatial sensitivities encoding a speed-up factor of 2 was used. A total of 70 axial slices parallel to the anterior commissure–posterior commissure line encompassing the entire brain in 51 directions with b = 900 s/mm^2^ was obtained, as well as 8 baseline scans with b_0_ = 0 s/mm^2^.

### VBM image processing and analysis

Voxel-based morphometry (VBM)^55^ was performed using Statistical Parametric Mapping 12 (SPM12, http://www.fil.ion.ucl.ac.uk/spm) and the Computational Anatomy Toolbox 12 (CAT12, http://www.neuro.uni-jena.de/cat/) implemented in MATLAB R2021a (Mathworks). After reorienting the T1-weighed images to define the anterior commissure as the origin, the T1-weighed images were segmented into GM, WM, and cerebrospinal fluid and normalized into a Montreal Neurologic Institute (MNI) space using CAT12. All normalized and modulated GM volumes were smoothed using an isotropic Gaussian kernel with a full width at half maximum of 6 mm. An absolute threshold masking of 0.1 was applied to restrict only voxels of gray matter volumes.

A whole-brain multiple regression analysis was done using the total scores of the COMOSWB as an independent variable and the TIV calculated using CAT12 as a covariate. Additionally, to control for possible confounding variables, age and sex were controlled for covariates. The significance threshold was set at a cluster-level of p < 0.05, FWE corrected. For further correlational analysis, the MarsBar tool box^56^ was used to extract mean gray matter volumes in a cluster that showed a significant correlation with the COMOSWB total scores for each participant.

### DTI image processing and analysis

The least-squares method was used with the Functional MRI of the Brain (FMRIB) Software Library (FSL; version 5.0; Oxford, UK; https://fsl.fmrib.ox.ac.uk/fsl/) to extract DTIs from DWI (approximate scan time, 17 min). All images were visually inspected for quality by an imaging specialist, and samples with no significant movement or other artifacts (e.g., warping) were chosen.

A whole-brain voxel-wise statistical analysis of fractional anisotropy (FA) data was performed using Tract-Based Spatial Statistics (TBSS) version 1.2, as provided in the FSL, according to the standard procedure^57^. The participants’ FA data were aligned in standard space (MNI 152 standard) and added and applied all transformed FA images to the original FA map, resulting in a standard-space version of the FA map. A mean FA image created by averaging all transformed FA images was thinned to obtain a mean FA skeleton representing the centers of WM tracts. The mean FA skeleton was controlled by setting a threshold value of FA > 0.2 (TBSS default) to contain only the major fiber bundles. Voxel-wise permutation-based nonparametric inference was performed on the skeletonized FA data using FSL Randomize version 2.1. General linear model regression analysis was performed, with 5000 random permutations, and the significance level was set at p < 0.05, corrected for the FWE rate. For further analysis, age and sex were controlled for as covariates. To avoid making an arbitrary choice of the cluster-forming threshold, a multiple comparison correction using threshold-free cluster enhancement (TFCE) was used, while preserving the sensitivity benefits of cluster-wise correction^58^. For further correlation analysis, the mean FA values of clusters that showed significant correlations with the total scores of the COMOSWB in the TBSS analysis were obtained using 3D slicer version 3.6^59^. In addition, voxel-wise statistical analysis further demonstrated the inter-relationship between significant clusters of the GM and WM using the TBSS. That is, after extracting the adjusted volume values of the significant GM regions correlated with SWB, TBSS voxel-wise correlation analysis was applied to explore the significant extracted clusters of the GM and their WM correlates.

### Statistical analysis

Pearson correlation analysis was performed to determine the relationship between WM FA values and GM volume extracted values of the significant clusters and other continuous variable such as the BAI, BDI-II, and WHOQOL-BREF. All statistical analyses except for voxel-wise analyses were performed using Statistical Package for the Social Sciences version 26.0 (IBM Corporation, Armonk, NY, USA). To solve the multiple comparison problem, an FDR correction was performed (q < 0.05).

### Ethical approval

All study procedures were reviewed and approved by the Institutional Review Board of CHA Bundang Medical Center in accordance with the latest version of the Declaration of Helsinki and principles of Good Clinical Practice.

### Informed consent

Informed consent was obtained from all individual participants included in the study.

## Data Availability

The datasets generated and analyzed during the current study are not publicly available due to legal or ethical restrictions that protect patients’ privacy and concent but available from the corresponding author on reasonable request.
